# Supplementary material for: Cronobacter spp. Isolated from Quick-Frozen Foods in China: Incidence, Genetic Characteristics, and Antibiotic Resistance
Source: Foods. 2023 Aug 11;12(16):3019. doi: 10.3390/foods12163019 (PMC10453260; doi:10.3390/foods12163019)
Supplement: Supplementary file 1 [file foods-12-03019-s001.zip › foods-2532181-supplementary.pdf]

Supplementary Material

Table

Table S1. The prevalence of *Cronobacter* isolated from frozen flour products in this study.

Frozen flour product	No. of samples	No. (%) of positive samples ($P<0.01$)		Positive sample contamination level (MPN/g)
Steamed stuffed buns	30	5	16.67%	1.29
Wonton	17	8	47.06%	0.80
Dumplings	165	81	49.09%	7.54
Total	212	94	44.34%	6.63

Table S2. The prevalence of *Cronobacter* isolated from frozen poultry in the present study.

Frozen poultry	No. of samples	No. (%) of positive samples ($P > 0.05$)		Positive sample contamination level (MPN/g)
Duck	5	0	0.00%	0.00
Chicken	234	9	3.85%	0.42
Total	239	9	3.77%	0.42

Table S3. The prevalence of *Cronobacter* isolated from frozen meat in this study.

Frozen meat	No. of samples	No. (%) of positive samples ($P<0.05$)		Positive sample contamination level (MPN/g)
Beef	36	0	0.00%	0.00
Mutton	67	2	2.99%	0.26
Pork	22	3	13.64%	0.15
Total	125	5	4.00%	0.22

Table S4. The prevalence of *Cronobacter* from quick-frozen food samples in every sampling city.

Cities	No. of samples	No. (%) of positive samples ($P > 0.05$)	
	SouthChina		
Guangzhou	61	7	11.48%
Shenzhen	14	4	28.57%
Shaoguan	13	3	23.08%
Zhanjiang	14	2	14.29%
Shantou	13	2	15.38%
Heyuan	12	3	25.00%
Haikou	12	3	25.00%
Sanya	13	3	23.08%
Beihai	14	4	28.57%
Nanning	13	2	15.38%
Macao	14	0	0.00%
HongKong	14	2	14.29%
	EastChina		
Fuzhou	13	3	23.08%
Xiamen	14	4	28.57%
Shanghai	14	6	42.86%
Hefei	13	5	38.46%
Nanchang	14	4	28.57%
Nanjing	14	1	7.14%
Hangzhou	14	4	28.57%
Jinan	14	2	14.29%

SouthwestChina			
Chengdu	14	3	21.43%
Kunming	14	1	7.14%
Lhasa	13	1	7.69%
Guiyang	14	2	14.29%
NortheastChina			
Harbin	14	4	28.57%
Changchun	14	1	7.14%
Shenyang	14	2	14.29%
NorthChina			
Taiyuan	13	2	15.38%
Hohhot	14	3	21.43%
Shijiazhuang	14	2	14.29%
Beijng	14	2	14.29%
CentralChina			
Zhengzhou	14	3	21.43%
Changsha	14	2	14.29%
Wuhan	13	2	15.38%
NorthwestChina			
Xian	14	5	35.71%
Yinchuan	14	2	14.29%
Lanzhou	11	3	27.27%
Urumqi	14	3	21.43%
Xining	13	1	7.69%

Table S5. Antimicrobial resistance rates of *Cronobacter* spp. identified in the present study.

Antimicrobial group	Antibiotic	Antimicrobial class[a] according to the WHO	No. (%) of *Cronobacter* spp. (n =154)		
			Resistant	Intermediate	Susceptible
Penicillins	Ampicillin (AMP, 10 μg)	CI	2 (1.30%)	0 (0.00%)	152 (98.70%)
	Ampicillin/sulbactam (SAM, 10 μg)	CI	1 (0.65%)	1 (0.65%)	152 (98.70%)
	Cefepime (FEP, 30 μg)	CI	0(0.00%)	0 (0.00%)	154 (100.00%)
	Ceftriaxone (CRO, 30 μg)	CI	2 (1.30%)	0 (0.00%)	152 (98.70%)
	Cefazolin (KZ, 30 μg)	HI	2 (1.30%)	18 (11.69%)	134 (87.01%)
	Cephalothin (KF, 30 μg)	HI	100 (64.94%)	42 (27.27%)	12 (7.79%)
Aminoglycosides	Gentamicin (CN, 10 μg)	CI	1 (0.65%)	1 (0.65%)	152 (98.70%)
	Tobramycin (TOB, 10 μg)	CI	0 (0.65%)	0 (0.00%)	154 (100.00%)
	Amikacin (AMK, 30 μg)	CI	1 (0.65%)	1 (0.65%)	152 (98.70%)
Quinolones	Ciprofloxacin (CIP, 5μg)	CI	0 (0.00%)	0 (0.00%)	154 (100.00%)
Carbapenems	Imipenem (IPM, 10 μg)	CI	0 (0.00%)	0 (0.00%)	154 (100%)
Sulfonamides	Trimethoprim/ sulfameth-oxazole (SXT, 25 μg)	HI	0 (0.00%)	1 (0.65%)	153 (99.35%)
Monobactams	Aztreonam (ATM, 30 μg)	HI	1 (0.65%)	0 (0.00%)	153 (99.35%)
Amoxicil-lin/clavulanic	Amoxicillin-clavulanic acid (AMC, 30 μg)	CI	0 (0.00%)	2 (1.30%)	152 (98.70%)
Amphenicols	Chloramphenicol (C, 30 μg)	HI	2 (1.30%)	1 (0.65%)	151 (98.05%)
Tetracyclines	Tetracycline (TE, 30 μg)	HI	3 (1.95%)	0 (0.00%)	151 (98.05%)

Figure

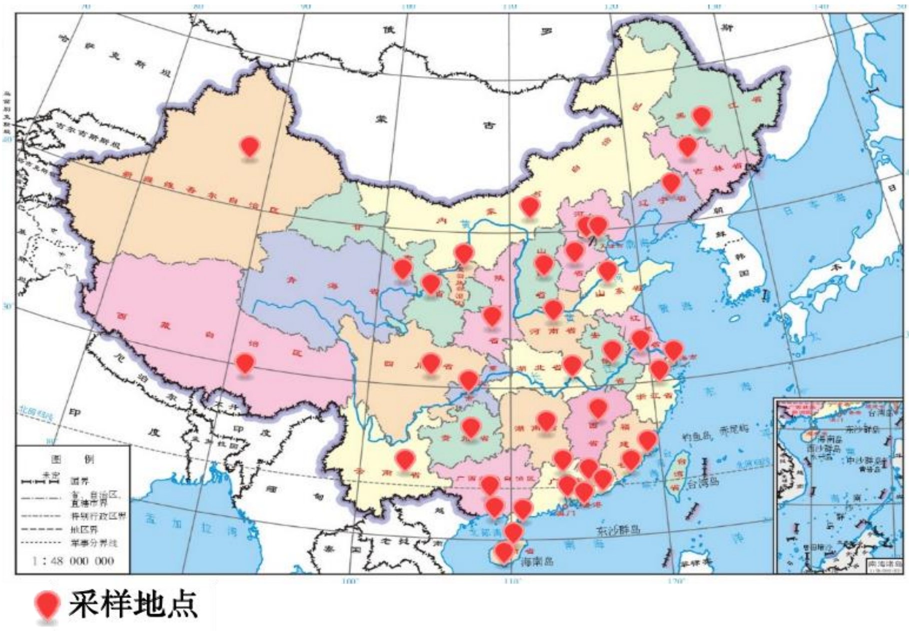

Figure S1. The locations of the sampling sites, including 39 cities, for this study in China.
